# Long COVID: a long road ahead

**DOI:** 10.1093/oxfimm/iqaf010

**Published:** 2025-12-13

**Authors:** Svetlana Blitshteyn

**Affiliations:** Department of Neurology, Jacobs School of Medicine and Biomedical Sciences, University at Buffalo, Buffalo, NY, USA; Dysautonomia Clinic, Williamsville, NY, USA

**Keywords:** Long COVID, autonomic dysfunction, dysautonomia, chronic fatigue syndrome/myalgic encephalomyelitis, public health, brain health, neurology

## Abstract

The SARS-CoV-2 pandemic caused an estimated 400 million people worldwide to experience Long COVID and post-COVID complications leading to significant chronic illness and disability with its devastating physical, societal and economic consequences. Since post-acute infectious syndromes have not been given adequate consideration prior to the pandemic, many millions of people with Long COVID worldwide have been left disabled as currently available therapies are largely symptomatic and only partially effective. A case of a previously healthy woman with Long COVID and post-COVID autonomic dysfunction and myalgic encephalomyelitis/chronic fatigue syndrome (ME/CFS) is presented here from the perspective of a physician-patient relationship and a broader context of medical care and public health. Immunologic and autonomic mechanistic factors and therapies as these relate to Long COVID are highlighted. Complexities and issues pertaining to patient care, public health and education of neurologists and other specialists regarding Long COVID, dysautonomia and ME/CFS diagnosis and treatment are discussed, in conjunction with the need to develop and diversify effective therapies for people living with these highly disabling conditions.

## Introduction

Long COVID refers to a diverse spectrum of symptoms, manifestations, end-organ damage, and post-COVID conditions that result in enormous disability and raise critical issues in how we approach the clinical care of patients with complex and disabling conditions. Physician-patient relationship and communication, available treatment options, immunologic and autonomic pathophysiologies of Long COVID, public health issues and brain health initiative that should include training of physicians competent to care for patients with Long COVID are discussed in this perspective. An illustrative case of a patient with Long COVID is presented, which serves as a foundation for other aspects of Long COVID, including the need for better healthcare, scientific and clinical infrastructure and, ultimately, a more effective therapeutic pathway.

## Long COVID: the patient

The patient was in her late thirties and was previously healthy and athletic, without past medical history. She had a thriving career and was an active mother to two young children. She contracted COVID-19, which resulted in a mild infection; however, following the infection, she never recovered. She developed severe fatigue, dizziness, orthostatic intolerance, post-exertional malaise and cognitive impairment, which forced her to quit her job. Bewildered that her symptoms after COVID-19 are not going away, with life being completely interrupted and put on hold, she searched for help among numerous medical specialists in order to attack the symptoms head-on; but instead of improvement, the symptoms seemed to attack her. She became house-bound and, at times, bed-bound, able to stand only a few minutes at a time due to orthostatic intolerance. Sometimes she had difficulty standing long enough to brush her teeth. She described her experience as being a prisoner in her own body and a far cry from her previously healthy and athletic self.

I saw her via telehealth, with most of the visits taking place with the patient lying in her bed in a dark bedroom with window shades shut and her eyes closed. She appeared pale and fatigued during many of our encounters. She told me that her world was shattered as the rest of the world moved on without her. In a soft voice to conserve her energy, she provided her medical history and updates on treatment. The history and course of illness were similar to hundreds of other patients with Long COVID, myalgic encephalomyelitis/chronic fatigue syndrome (ME/CFS) and dysautonomia that I have seen before. Every patient with these complex and disabling conditions had the same goal: to get better and return to their former self when they were healthy. Almost none of my patients had anhedonia, distorted thinking, abnormal behavior or pathological attitude to suggest a psychiatric disorder as the culprit of their disability. Almost every patient had unrelenting fatigue, physical and mental exhaustion and inability to engage in even the smallest of daily tasks without worsening their symptoms in a post-exertional malaise.

## Long COVID: autonomic dysfunction and ME/CFS

After acute SARS-CoV-2 infection, an estimated 400 million people globally and 17 million, or 6.8% of Americans, based on Household Pulse Survey, experienced Long COVID symptoms, such as fatigue, cognitive impairment, body pain, headache, dizziness, sleep disturbance and a myriad of other symptoms and signs spanning multiple body systems [1, 2]. At least half of the patients qualify for a diagnosis of ME/CFS based on its clinical criteria [3]. Despite a long list of supplements and medication trials, as well as non-pharmacologic therapies, both conventional and alternative, my patient, like millions of others, was still sick and unable to function. When traditional medical system has no answers for people with complex chronic disorders—a common theme that has permeated the medical establishment—patients seek answers outside of it, an act fueled by self-advocacy, desperation and a strong desire to get better.

Traditionally, many people like my patients were told by their physician that the nature of their symptoms is psychological, psychosomatic, functional, rooted in anxiety, past trauma, faulty coping mechanisms or impaired self-agency. These theories—most unproven—have yielded very little in terms of effective therapies for patients. Examining the evidence on Long COVID, we acknowledge that Long COVID is not a mental health condition and is not caused by biopsychosocial, socioeconomic or psychological causes [4].

As clinicians, our job is to diagnose correctly, identify most disabling symptoms and establish a treatment plan that targets these symptoms and their possible underlying pathophysiology. For patients with disabling fatigue and orthostatic intolerance, finding the right combination of medications and non-pharmacologic therapies to get patients out of bed is the key to improvement. As a neurologist specializing in autonomic disorders, bed-bound and house-bound patients with severe symptoms have always presented a special challenge to find treatment that would enable them to defy the effects of gravity by improving their dysfunctional autonomic nervous system (ANS). Nowhere in medicine does the interface between the brain, the ANS and the cardiovascular and immunologic functions matters as much as it does in autonomic disorders [5]. The brain and the heart, connected via the ANS, must coordinate in perfect synchrony to keep us vertical and avoid orthostasis: if this synchrony is jeopardized, an imbalance between the sympathetic and parasympathetic nervous system ensues—a key feature of autonomic dysfunction as part of Long COVID and many other systemic diseases associated with dysautonomia [6]. The autonomic and immunologic systems are intertwined via inflammatory reflex and other yet unidentified pathways involving the central and peripheral nervous systems [7]. While much in the realm of autonomic modulation of the immune system and immune regulation of the autonomic nervous system remains under investigation, there is emerging evidence that sympathetic overactivity is associated with pro-inflammatory process and that parasympathetic activation has anti-inflammatory properties [8, 9]. These developing concepts are highly relevant to the pathophysiology of Long COVID and all post-acute infectious syndromes.

Unfortunately, a multitude of therapies that we tried with the patient didn’t yield significant improvement that we had hoped for. The treatment trials felt like a failure despite my approach to complex chronic disorders, which is to be aggressive with the available therapies and to think outside the box while respecting the science and art of the box. Still… none of the beta blockers, volume expanders, vasoconstrictors, stimulants, antidepressants, antivirals and anti-inflammatories that we tried made a difference in the profound fatigue, cognitive impairment and post-exertional malaise that my patient experienced. Although both of us were highly motivated to find successful treatment, nothing seemed to work despite our best efforts and our collective enthusiasm for thinking and doing things outside of the proverbial box.

“Will I ever get better?” is the ultimate question that begs for any rays of hope amidst desperation and insurmountable suffering. Complex and disabling chronic illness that has no effective treatment is a force to reckon with: it destroys lives, decimates dreams and demoralizes the most psychologically strong individuals.

“Yes, you will.” I answered cautiously when the patient asked me that question after multiple treatment failures. As physicians, our job is also to keep hope alive when treatments fail—hope that’s rooted in clinical experience and science that promises new treatments with ongoing clinical trials, not false hope or wishful thinking.

## Long COVID: physician-patient communication

How physicians communicate with patients and their families is essential to what the patient absorbs and internalizes about their illness, how they view their future and what they tell themselves about their altered life and hope to get better [10]. The hope to get better is the key to maintaining mental health and not letting it devolve into an existential crisis. As physicians, we are primed to treat to improve outcomes; we aspire to functional restoration. If we fail to do so, the knee-jerk response for many physicians is to look for reasons we fail. When our own ego supersedes our humility and compassion, we become defensive and often blame the patient when they don’t get better: after all, their diagnostic tests look “normal,” the patients are relatively young and do not appear to have any malignant or known degenerative processes that can make them as sick and dysfunctional as they claim to be. Then to absolve ourselves of responsibility and self-blame, we become suspicious: what if the patient is not sick or not “that” sick? What if this is not physical and the patient is “just” mentally or psychologically unwell? What if they “fear” life outside of their bedroom, what if they “fear” sunlight, fresh air and moving their bodies in space and time? What if they fear and avoid the actual living? Is it fear and avoidance that drives their dysfunction, or is it pathologic alterations that affect their physical and mental energy? By all measures that are written in medical textbooks, they should not be so impaired, debilitated and dysfunctional… We wonder, we have our fears, our suspicions, our doubts… We turn to psychiatry and psychology to help us, to help us help them, to help decipher where the brain begins and the mind ends.

I always tell my patients that the brain is not separate from the body. Systemic processes and abnormal pathobiology will often translate into neuropsychiatric manifestations of systemic disease. Ignoring the underlying systemic etiology of neurologic and psychiatric symptoms will not result in meaningful and long-lasting management strategies: it’s one of the major reasons why all forms of psychological, behavior and physical therapy is often ineffective in a majority of patients with post-acute infectious syndromes [11].

## Long COVID: public health

Historically, patients with post-infectious syndromes have been misdiagnosed with somatoform or psychological disorders due to the absence of abnormalities on routine testing. Recent evidence demonstrates physiological mechanisms underlying these conditions, underscoring the need to avoid misattribution and ensure appropriate care. These mechanisms include chronic inflammation, immune dysregulation, viral persistence, autonomic dysfunction and other pathophysiologic factors underlying post-acute infectious syndromes [12]. Autonomic dysfunction—a major piece of the Long COVID puzzle—is when we have to utilize our clinical skills with history, neurologic exam, a 10-minute stand test, a tilt table test and a skin punch biopsy as our tools for diagnosis [13]. My patient had the means to find and afford a multitude of specialists and treatments, but many patients are not so fortunate. In an ironic twist of fate, a previously neglected field of post-acute infectious syndromes received the most attention, advocacy, and the National Institutes of Health (NIH) research funding than ever before [14] - thanks to COVID pandemic-generated silver lining that none of us anticipated, but all of us specializing in this field were hoping for.

Long COVID is a major public health issue with long-lasting adverse health effects and impact on the individual and public health [15]. Besides cognitive, autonomic and neurologic sequelae, post-COVID adverse health outcomes affecting almost every organ and body system have been identified [16]. Emerging evidence suggests that the risk of Long COVID and post-COVID complications, including all-cause mortality, is increased with each subsequent COVID infection [17]. Many known and unknown adverse effects of SARS-CoV-2, including repeated infections on the developing brain of children and adolescents, its carcinogenic effects and deleterious effects on aging and neurodegeneration, remain [18–20].

The SARS-CoV-2 pandemic is not over, with other possible pandemics looming around the corner. As we find ourselves amidst environmental and economic challenges, as well as pandemic fatigue, a spike in Long COVID and post-acute infectious chronic illness is expected.

## Long COVID: brain health

Autonomic and autoimmune neurology is at the forefront of groundbreaking discoveries in the interplay between the brain and the immune system. Beyond familial dysautonomia, neurogenic orthostatic hypotension and demyelinating disorders, there is a broad range of neuroimmune conditions that affect the brain, the peripheral nervous system, including small fiber and autonomic neuropathy and ganglionopathy, the immune system, the cardiovascular system, the gastrointestinal system, the genitourinary system, the pulmonary system, the connective tissue, the endothelium, the mitochondria and the epigenetic phenotypes. These conditions include post-acute infectious syndromes, ME/CFS and dysautonomia—a common comorbidity of systemic disease [21]. Education and training of current and future neurologists must include new topics that were previously not a part of the learning curriculum, such as how to recognize, diagnose and treat dysautonomia, ME/CFS, Long COVID and other post-acute infectious syndromes, most of which involve significant neurologic, autonomic and systemic manifestations [5, 21]. To omit these disorders from clinical neurology would mean to exclude a large subset of patient population experiencing neurologic manifestations and syndromes from obtaining appropriate healthcare.

The recently launched initiative “Brain Health for All” by the American Academy of Neurology states that neurologists are uniquely positioned to serve as specialists in brain health and to advance the newly evolving field of preventive neurology, which aims to identify individuals at high risk of brain disorders and other neurologic conditions and offer strategies to mitigate disease emergence or progression [22]. The initiative calls for neurologists and other specialists to accomplish the goal of reducing the burden of neurologic disorders and achieving optimal brain health for all as a public health issue [22]. The goal is to accelerate scientific discovery in brain health through cross-disciplinary collaboration and to optimize brain health through integration of preventive care practices [22]. This initiative cannot succeed without including dysautonomia, ME/CFS, Long COVID and other post-acute infectious syndromes on the list of priorities and major factors of neurologic disease burden and disability. To this end, the brain health curriculum must include a structured and contemporary module on dysautonomia, ME/CFS and Long COVID as part of the neurology residency training and continuous medical education.

## Long COVID: therapeutic toolbox

Finally, and most importantly, to combat these complex, disabling and highly prevalent disorders, we must develop a diverse assortment of effective pharmacologic and non-pharmacologic therapies for Long COVID in this long road ahead of us. These therapies cannot exclusively consist of exercise, psychotherapy or cognitive training—the widely utilized treatment modalities that are offered to most patients despite their marginal benefits or a lack of efficacy [23, 24]. Given the diversity and broad range of phenotypes, treatment for Long COVID must be individualized and patient-centered, administered by a multidisciplinary team consisting of experts and experienced medical and allied health professionals. The arsenal of therapies may include immunomodulatory, anti-inflammatory, antiviral and other new and repurposed therapies that should be made accessible and affordable: there must be an easy and time-sensitive path for patients to obtain these prescribed treatments without needing to appeal denials or engage in protracted bureaucratic process. New diagnostic tests that include serum immunologic and autoimmune tests, autonomic tests and specialized neuroimaging modalities, as well as wearable devices that measure cerebral perfusion and vital signs, should be readily available to clinicians and patients as part of routine patient care. Medical professionals of all specialties must get training in managing complex patients with Long COVID, ME/CFS and dysautonomia.

My patient finally experienced some improvement in her symptoms after receiving intravenous immunoglobulin (IVIG) – an immunomodulatory therapy consisting of pooled plasma from multiple blood donors. IVIG has been used for over 50 years for neuromuscular disorders, autoimmune diseases and immunodeficiencies, but its benefits have been also noted in patients with POTS, other common autonomic disorders and Long COVID [25]. IVIG may have anti-inflammatory properties, which could be why my patient and others with Long COVID improve. However, this therapy is expensive and heavily regulated in the United States, with coverage provided only for a handful of selected conditions, which excludes most patients with Long COVID, ME/CFS and dysautonomia. My patient’s ability to gain access to IVIG was an exception: normally, patients are unable to try this potentially effective therapy despite its possible benefit [25]. The NIH-RECOVER Autonomic trial is currently conducting a multi-center randomized controlled trial of IVIG for post-COVID POTS [26] – an important trial that could change the therapeutic landscape for Long COVID and other post-acute infectious syndromes for millions of patients who have been waiting to get better for many years. I too can’t wait to get these new therapies into my toolbox.

## Conclusion

With SARS-CoV-2 pandemic and resultant Long COVID, there is an urgent need to establish an infrastructure and framework for public health, research, diagnosis and treatment of post-acute infectious syndromes. Many millions of people with Long COVID are left with complex and disabling chronic illness that has limited therapeutic options to date. Given the interplay between autonomic and immunologic pathophysiologic mechanisms of post-acute infectious syndromes, targeted evidence-based therapies to address these mechanistic factors are needed as well as education and training of physicians to gain competence in managing these complex patients.

## Data Availability

No new data were generated or analysed in support of this research.
